# Ultrasonography of abdominal muscles: Differential diagnosis of late-onset Pompe disease and myotonic dystrophy type 1

**DOI:** 10.3389/fneur.2022.944464

**Published:** 2022-09-06

**Authors:** Pei-Chen Hsieh, Chun-Wei Chang, Long-Sun Ro, Chin-Chang Huang, Jia-En Chi, Hung-Chou Kuo

**Affiliations:** ^1^Department of Neurology, Linkou Chang Gung Memorial Hospital, College of Medicine, Chang Gung University, Taoyuan, Taiwan; ^2^College of Medicine, Chang Gung University, Taoyuan, Taiwan; ^3^Department of Orthopedic Surgery, Bone and Joint Research Center, Linkou Chang Gung Memorial Hospital, College of Medicine, Chang Gung University, Taoyuan, Taiwan

**Keywords:** late-onset Pompe disease, myotonic dystrophy type 1, muscle ultrasonography, abdominal muscle, trunk function

## Abstract

**Introduction:**

Axial muscles are involved earlier and to a greater extent in late-onset Pompe disease (LOPD) than in myotonic muscular dystrophy type 1 (DM1). We aimed to evaluate abdominal muscles in LOPD compared in DM1 using muscle ultrasonography.

**Methods:**

Patients with LOPD (*n* = 3), DM1 (*n* = 10), and age- and gender-matched healthy subjects (*n* = 34) were enrolled for muscle ultrasonography. Patients with LOPD and DM1 were 20 to 59 years of age with a disease duration ranging between 7 and 30 years. A multifrequency linear transducer was used to evaluate quality and thickness in the abdominal muscles and extremities.

**Results:**

The quantitative muscle echo score revealed a higher Z score in abdominal muscles in Patients with LOPD (scores were relatively normal for the biceps and flexor digitorum groups). Patients with LOPD had significantly lower abdominal muscle thickness than patients with DM1. Abdominal muscle strength was significantly correlated with the muscle echogenicity, trunk impairment scale, and trunk control test. The extremities' sum score was correlated with the total Medical Research Council score.

**Discussion:**

The increased quantitative muscle score in abdominal muscles, sparing the biceps and flexor digitorum groups, may offer differential diagnosis between LOPD and DM1. Ultrasound can easily access abdominal muscles and investigate muscle echogenicity and thickness. A quantitative approach using muscle echogenicity rather than muscle thickness may provide a greater correlation with trunk muscle function.

## Introduction

Pompe disease is a rare glycogen storage disease (1). This autosomal recessive disease is caused by acid alpha-glucosidase (GAA) deficiency (1, 2) resulting in progressive accumulation of alpha-glucosidase substrate in lysosomes and cytoplasm. Pompe disease manifests clinically across a wide range of severity. A core feature of late-onset Pompe disease (LOPD) is early involvement of trunk muscles, resulting in trunk deformity and difficulty rolling in bed (3). MRI using T1-weighted sequences identified fatty replacement in abdominal muscles, paraspinal muscles, and gluteal muscles (4, 5). Muscle ultrasound has been used as a diagnostic tool to identify muscle inflammation, fatty infiltration, and fibrosis. It is a cost-effective, efficient, and safe method for the detection of subclinical or clinical changes in neuromuscular disorders (6, 7). Abdominal muscles are relatively easy to visualize using skeletal muscle ultrasonography. By contrast, myotonic dystrophy type 1 (DM1) is caused by CTG nucleotide repeat expansion in the myotonic dystrophy protein kinase (DMPK) gene (8, 9). The typical manifestations of DM1 are progressively distal to proximal weakness with atrophy of the temporalis muscle. Spine deformity and abdominal muscle involvement may occur in late-stage DM1 (8, 10).

In clinical practice, the Medical Research Council (MRC) score is used to evaluate muscle strength, particularly in the extremities; however, there is no scoring system to evaluate patient's trunk function in patients with myopathy. The trunk muscles, including the abdominal muscles, are the largest muscle groups in the human body, which help to maintain sitting and standing posture, maintain balance, and smoothly connect movement between different postures (11). Poor trunk function may cause an increased falling risk, a decreased daily activity, and decreased upper and lower limb functional movements (11). In addition, the fat replacement of back, abdominal, and deep trunk muscles have been found to be correlated with poor respiratory function in LOPD (12). In recent studies, trunk impairment score (TIS) and trunk control test (TCT) have been used to assess static and dynamic trunk muscle function in patients with stroke (11, 13). These are easy and reliable measurements of trunk performance in patients with neuromuscular disease (14–17).

Herein, we compare ultrasonography images between patients with LOPD and patients with DM1 to determine if a correlation exists in total MRC score, abdominal muscle strength, TCT, and TIS score with muscle echogenicity and thickness.

## Methods

### Patients and their clinical data

Patients diagnosed with LOPD or DM1 in a medical center between January 2014 and December 2018 were included. A detailed history, clinical features, neurologic examination, genetic and biochemical tests, and electrophysiological studies were collected. A diagnosis was made according to clinical characteristics, pathological findings, and genetic analysis. The genetic test for LOPD was performed using extracted genomic deoxyribonucleic acid (gDNA) from peripheral blood. All GAA exons and intron-exon boundaries were amplified using PCR, and the PCR products were sequenced by Sanger sequencing. Sequences were compared with the GAA reference DNA sequence (NM_000152) to identify mutations. The complementary DNA (cDNA) was numbered with +1 corresponding to the A of the ATG translation initiation codon and with codon 1 as the initiation codon. The genetic test in patients with DM1 was performed with two methods. Southern blotting method was performed in 7 patients, while triplet-primed PCR was performed in 3 patients (18–20).

The study protocol was approved by the Institutional Review Boards of Chang Gung Memorial Hospital (ethical license No: 202101058B0). Informed consent was obtained from study participants.

### Ultrasound assessment protocol and evaluation of motor function

Patients with LOPD and DM1 were recruited (age at examination: 20–70 years; disease duration: 7–30 years). All patients received muscle ultrasonography by 2 examiners using neuromuscular ultrasonography. Muscle thickness and echogenicity were assessed using a multifrequency linear transducer (UP200, BenQ Medical Technology, Corp., Taipei, Taiwan). The ultrasound device frequency was automatically adjusted to the higher frequency with a gain of 53 dB. Dynamic scans were set at 58 dB in all targeted muscles. Zooming in was avoided to maintain consistency in all measurements. Depth was dependent on muscle thickness and the distance from skin tissue. Muscle thickness and echogenicity were measured on cross-sectional images using the ultrasonography distance measurement function. All the patients were evaluated in the supine position with minimal pressure to avoid muscle compression. Intra- and inter-rater reliabilities were evaluated in all of the 10 candidates. To demonstrate reliability, each selected muscle was measured at least 2 times on separate days by one neurologist, with 2 years of neuromuscular ultrasonography experience, and a technician, with 1 year of neuromuscular ultrasonography experience. The interval between the measurements was at least 2 days (the longest interval was 5 days) and the raters were blinded to previous results (Supplementary Table 1).

The muscle scans were performed bilaterally according to standards in the following muscles: biceps brachii (BB) (mid-arm), triceps (mid-arm), flexor digitorum superficialis/flexor digitorum profundus (FDS/FDP) (in the mid-forearm), rectus femoris (RF), tibialis anterior (TA), rectus abdominis (RA), external oblique (EO), internal oblique (IO), and transversus abdominis muscles (TrA). The linear ultrasound probe was placed 3 cm lateral to the umbilicus for the RA. For the EO/IO/TrA muscle groups, the probe was placed at 3 cm medial to the mid-axillary line horizontally to umbilicus line (13, 21). The gray-scale analysis in the region of interest was performed for all sampled muscles. A mean gray-scale value for each image was obtained and compared to a muscle-specific reference from healthy controls (demonstrated as Z score) (21, 22). The measurements were corrected for age and gender. The ultrasonography setting of healthy control groups were consistent with patient groups. Abnormal echogenicity was defined as a Z score >2 (21, 22).

The Medical Research Council (MRC) sum score was calculated using the total of the deltoid, biceps brachii, triceps, wrist flexor/extensor groups, finger flexors, iliopsoas, gluteus, quadriceps, hamstrings, gastrocnemius, and tibialis anterior muscles. Abdominal muscle strength was graded in the supine position: grade 1: only feel muscle contraction; grade 2: Pelvic posterior tilt; grade 3: bilateral hand could touch the knee; grade 4: full trunk curl with arms folded over chest; and grade 5: full trunk curl with arms behind the neck (Supplementary Table 2). Abdominal muscle strength was scored with an adapted Medical Research Council (MRC) scoring (Supplementary Table 2) (10). TCT and TIS were used to evaluate all the trunk motor functions. The TCT score has 4 items with a minimum score of 0 and a maximum score of 100. The total score for TIS ranged from 0 to 23 with 3 major functions evaluated: static sitting balance, dynamic sitting balance, and coordination. Each group included at least five healthy controls to analyze average muscle thickness, abdominal muscle strength, and MRC sum score (results are shown in Supplementary Table 3).

### Statistical analysis

Clinical and laboratory data are presented with descriptive statistics. The Mann–Whitney U-test was used to evaluate differences in demographic data and Z score of muscle echogenicity between LOPD and DM1. The Spearman ρ correlation coefficient test was performed to analyze abdominal muscle strength, TCT, TIS, trunk muscles echogenicity, and thickness. The SPSS version 22 (International Business Machines Corporation, USA) software was used for all statistical analyses.

## Results

### Demographic data and clinical manifestation

The demographic data of patients with LOPD and DM1 are reported in Table 1. Patient age ranged from 24 to 31 and 23 to 59 years for patients with LOPD and patients with DM1, respectively. Three men with LOPD and 7 women and 3 men with DM1 were evaluated. The disease diagnoses were confirmed with a genetic analysis, and the abdominal muscle strength (MMT), MRC, TIS, and TCT are shown in Table 1. The LOPD group revealed lower TCT and TIS than the DM1 group, but there was no significant difference between two groups.

**Table 1:** Demographic and functional data in late-onset Pompe disease and Myotonic dystrophy type 1.

	**Gender/age (y)**	**Genetic mutation**	**Disease Duration (yr)**	**Wheelchair dependent**	**Abdominal muscle strength (0–5)**	**MRC sum score (0–120)**	**TCT (0–100)**	**TIS Total score (0–30)**
**LOPD Patients**								
1	M/24	GAA c.2238G>C, homozygous	12	N	1	114	87.5	22
2	M/30	GAA c.2228A>C, homozygous	13	Y	1	100	50	16
3	M/31	GAA c.2228A>C, homozygous	12	N	1	110	62.5	20
**DM1 patients**								
1	F/59	266 of CTG triplet repeats	20	Y	2	90	87.5	27
2	F/32	1,000 of CTG triplet repeats	30	N	2	98	62.5	23
3	M/56	>300 of CTG triplet repeats	24	Y	1	40	50	17
4	F/57	>300 of CTG triplet repeats	10	Y	1	58	50	12
5	F/35	>300 of CTG triplet repeats	7	N	5	120	100	30
6	F/54	162 of CTG triplet repeats	12	N	2	90	87.5	28
7	F/54	126 of CTG triplet repeats	14	N	3	118	100	28
8	M/29	762 of CTG triplet repeats	13	N	5	118	100	28
9	M/38	762 of CTG triplet repeats	10	N	2	112	100	28
10	F/23	762 of CTG triplet repeats	13	N	5	118	100	30

LOPD, late-onset Pompe disease; DM1, myotonic muscular dystrophy type 1; MRC, medical research council; TCT, trunk control test; TIS, trunk impairment score; Y, Yes; N, No.

### Ultrasonographic values for muscular echogenicity and thickness

**Figure 1:**
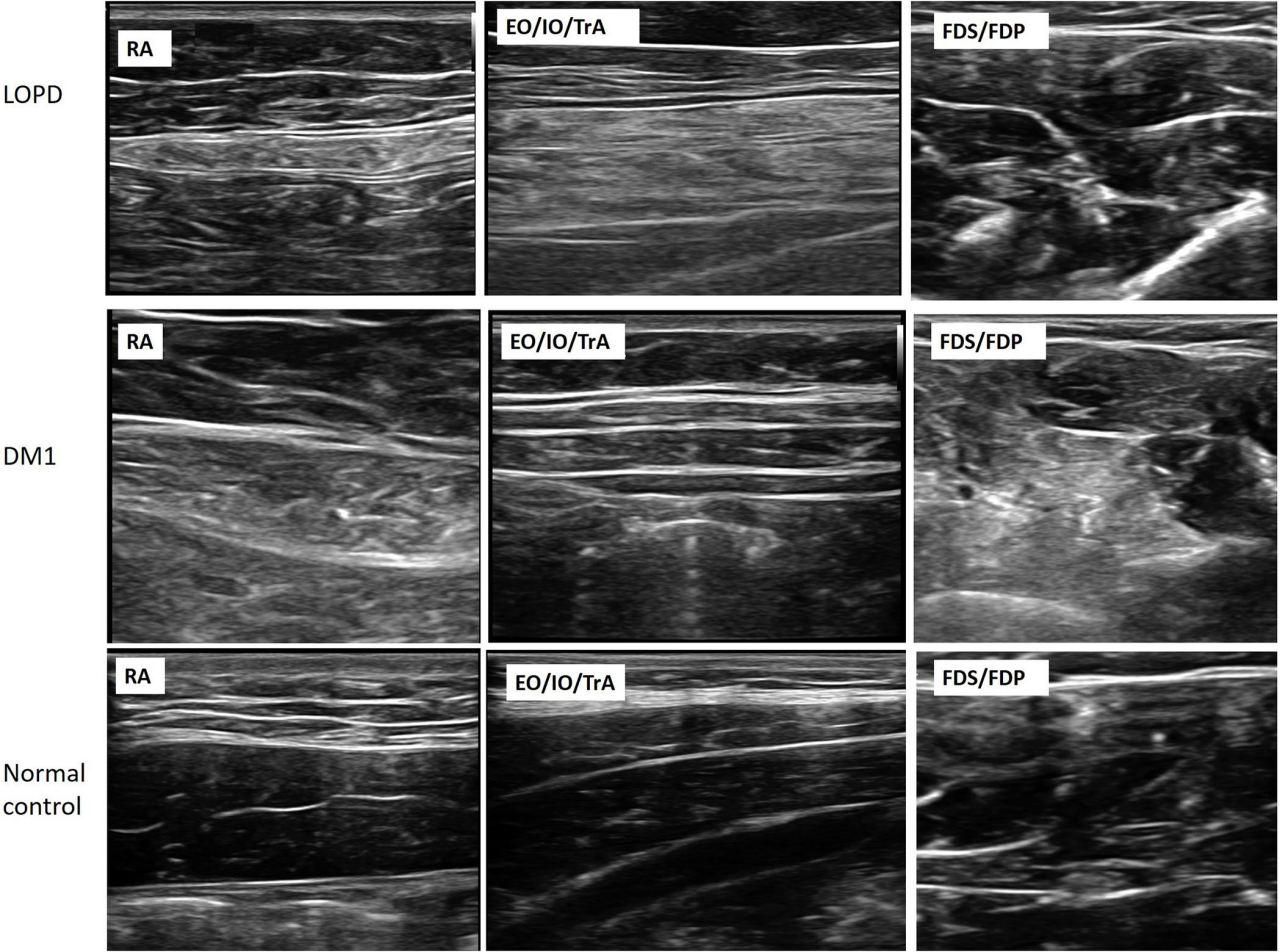
Muscle ultrasonography of RA, EO/IO/TrA, and FDS/FDP muscles in the patients with LOPD, DM1, and normal controls. The images demonstrate increasing muscle echogenicity and atrophy of trunk muscles and normal echogenicity of FDS/FDP muscles in the LOPD group. For the DM1 group, the images show mildly increasing echogenicity in trunk muscles and increasing echogenicity in FDS/FDP muscles. LOPD, Late-onset Pompe disease; DM1, Myotonic dystrophy type 1; HC, Healthy control; RA, Rectus abdominis; EO, External oblique; IO, Internal oblique; TrA, Transversus abdominis; FDS/FDP, flexor digitorum superficialis/flexor digitorum profundus.

Images from patients with LOPD revealed increased echogenicity and atrophy in RA and EO/IO/TrA muscles, and relatively normal appearance of muscles in the extremities. Atrophy and increased echogenicity were observed in distal parts of extremities, particularly in FDS/FDP and TA muscles, and mild increases in echogenicity in abdominal muscle groups in Patients with DM1 (Figure 1).

**Figure 2:**
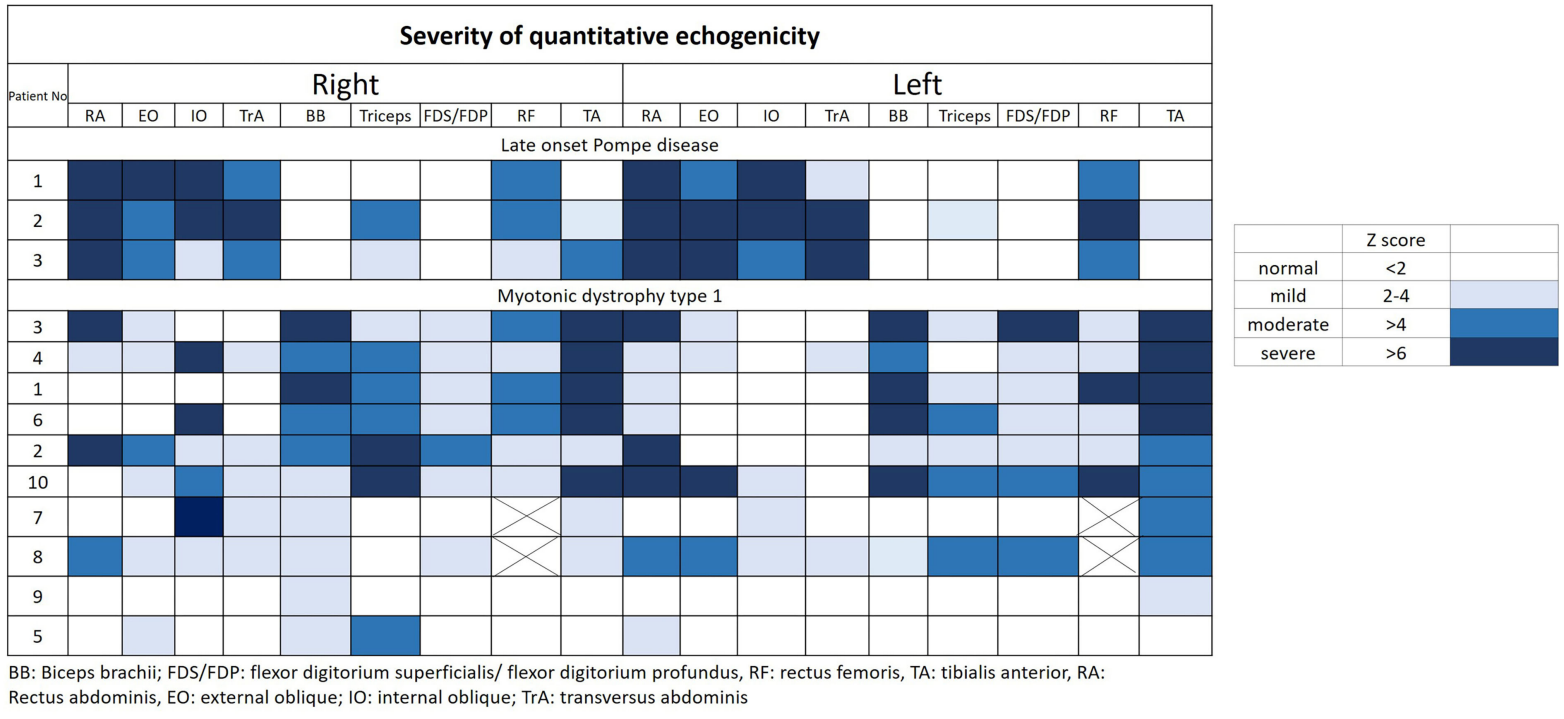
Quantitative echogenicity severity in patients with LOPD and DM1. LOPD, Late-onset Pompe disease; DM1, Myotonic dystrophy type 1.

The muscular ultrasonographic values of the patients with LOPD revealed moderate to severely increased echogenicity of most abdominal muscles (Z score >4). The severe involvement of abdominal muscles was 66.7% (16/24) in LOPD and 11.25% (9/80) in DM1 (Figure 2). There were significant differences in quantitative muscle echogenicity between patients with LOPD and patients with DM1 in bilateral RA, EO, and TrA, and left IO. The total trunk grading sum score revealed a statistically significant difference between the two groups. The total abdominal muscle thickness was significantly decreased in patients with LOPD after adjustment for age and gender (Table 2).

**Table 2:** Quantitative echogenicity and abdominal muscle thickness in muscular ultrasonography.

	**Late onset Pompe disease**	**Myotonic dystrophy 1**	***P*-value**
Age	28.33	43.7	0.112
Medical Research Council	108	96.2	0.937
Trunk Control Test	66.67	83.75	0.217
Trunk Impairment Score	19.33	25.30	0.112
**Muscle echogenicity (Right/Left; Z score)**
Rectus abdominis	10.63762/11.0496	3.062263/4.313832	0.028*/0.028*
External oblique	6.127369/9.966672	1.779355/2.912854	0.007**/0.028*
Internal oblique	7.610181/8.487429	4.408037/2.273784	0.287/0.014*
Transversus abdominis	6.656446/12.56095	3.008856/1.697471	0.049*/0.028*
Biceps	0.713116/1.110798	4.207454/4.058665	0.009**/0.1
Triceps	2.452227/0.746397	3.657584/3.339666	0.537/0.217
Flexor digitorium^†^	0.188559/-4.5199	2.433022/5.147085	0.036*/0.009**
Rectus femoris	3.893264/6.762521	3.168355/3.723121	0.833/0.183
Tibialis anterior	3.609776/1.715406	5.030613/6.112169	0.6/0.1
Modified Total sum score	44.33	38	0.937
Extremities Sum Score	16	24.12	0.194
Trunk Sum Score	28.33	14	0.007**
**Muscle thickness (Right/Left; Z score)**
Trunk muscle thickness	−4.46/-6.45	−1.27/−1.07	0.028*/0.028*

^†^,flexor digitorium superficialis and flexor digitorium profundus; Trunk Sum score: sum of quantitative severity score in trunk; Extremities Sum score: sum of quantitative severity score in extremities; Modified Total sum score: sum of quantitative severity score in all muscles.

^*^P < 0.05; ^**^P < 0.01.

The muscle echogenicity of BB muscles in all patients with LOPD was considered normal. The lower MRC score was associated with increased echogenicity in BB muscles of most patients with DM1, and there were also significant differences in right BB muscles between the two groups. In the triceps muscles, echogenicity was increased in most patients with DM1 and was relatively normal in patients with LOPD; however, there was no statistical difference between the two groups. The muscle echogenicity of the two groups was abnormal in RF muscles. In distal extremities, muscle echogenicity was relatively normal in the LOPD group, but significantly greater in FDS/FDP muscles and a trend of higher, but not statistically significant differences in TA muscle in the DM1 group (Table 2 and Figure 2).

**Figure 3:**
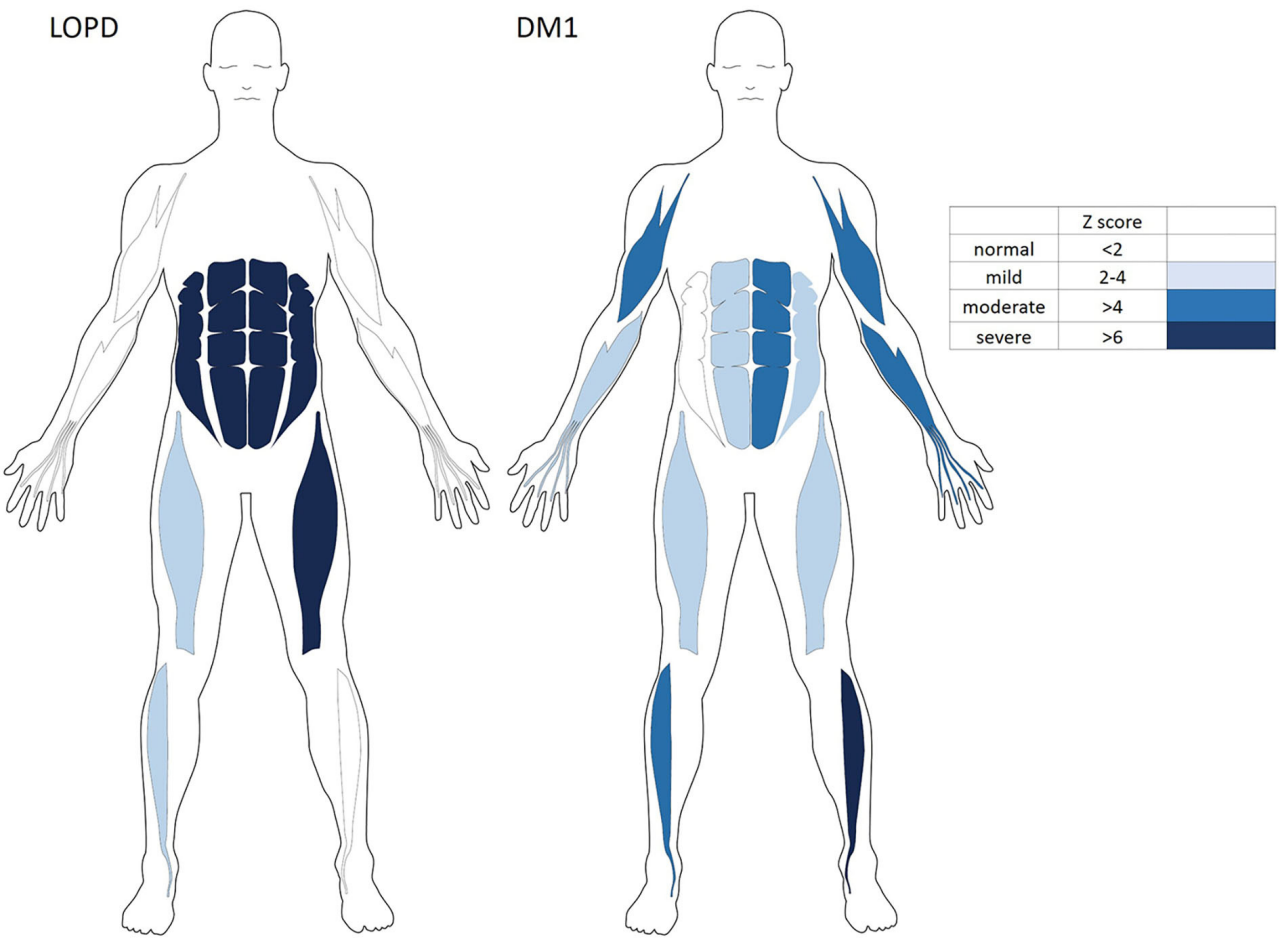
The variable severity and distribution of qualitative echogenicity in skeletal muscles of the LOPD and Patients with DM1. DM1, Myotonic dystrophy type 1; LOPD, Late-onset Pompe disease.

Muscle ultrasonography revealed to be abnormal in the abdominal muscles, mildly abnormal in rectus femoris and anterior tibialis muscles, as well as sparing in biceps and FDS/FDP muscles in the LOPD group. By contrast, there was less abnormal in abdominal muscles and more obvious in biceps, AT, and FDS/FDP involvement in the DM1 group (Figure 3).

### The correlation between ultrasonographic values and motor function of trunk muscles

**Figure 4:**
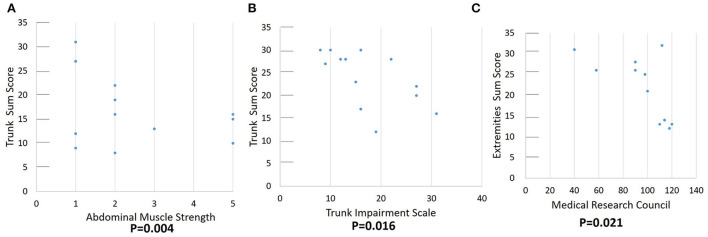
Correlation of function score and quantitative echogenicity severity of skeletal muscles. **(A,B)** demonstrate a significant correlation between total echo trunk sum score and abdominal muscle strength and trunk impairment scale. **(C)** demonstrates a significant correlation between MRC and total echo extremities sum score.

The correlation between the echogenicity and thickness of muscle ultrasonography and the trunk motor function, including abdominal muscle strength, MRC sum score, TIC, and TIS total scores are shown in Table 3. Muscle strength was correlated with echogenicity of RA, EO, and IO muscles. The total echo trunk sum score and MRC sum score were significantly correlated with abdominal muscle strength; there was no significant correlation with abdominal muscle thickness. The MRC score was not correlated with abdominal muscle echogenicity or thickness. The TCT score was correlated with EO and TrA echogenicity and highly correlated with MRC score and abdominal muscle strength. The TIS score was correlated with EO muscle, trunk sum score, MRC sum score, and abdominal muscle strength. The total echo trunk sum score was correlated with TIS and abdominal muscle strength (Figures 4A,B). The total echo extremities muscle sum score was significantly correlated with MRC score (Figure 4C).

**Table 3:** Correlation between function tests and muscle sonography of trunk muscles^‡^.

	**Abdominal muscle strength**	**MRC sum score**	**TCT total score**	**TIS total score**
**Abdominal muscle (R/L)**
***Rectus abdominis***
Thickness	0.052/−0.003	−0.114/−0.443	−0.227/−0.326	−0.022/−0.133
Echogenicity	−0.632*/−0.646*	−0.191/−0.205	−0.490/−0.469	−0.544/−0.520
***External oblique***
Thickness	0.003/−0.087	−0.014/−0.445	−0.140/−0.284	−0.056/−0.211
Echogenicity	−0.707**/−0.566*	−0.158/−0.185	−0.590*/−0.341	−0.639*/−0.394
***Internal oblique***
Thickness	0.313/0.103	−0.136/−0.104	−0.001/−0.109	0.220/0.061
Echogenicity	−0.392/−0.571*	−0.122/−0.064	−0.238/−0.324	−0.322/−0.500
***Transversus abdominis***
Thickness	0.251/−0.032	0.003/−0.279	0.132/−0.198	0.356/−0.167
Echogenicity	−0.407/−0.580	0.127/−0.371	−0.226/−0.564*	−0.428/−0.500
**Total thickness (R/L)**	0.312/0.289	−0.172/−0.097	0.009/0.112	0.222/0.206
**Trunk Sum core**	−0.732**	−0.172	−0.49	−0.652*
**MRC sum score**	0.686*		0.813**	0.727**
**Abdominal Muscle strength**		0.686*	0.859**	0.948**

MRC, medical research council; TCT, trunk control test; TIS, trunk impairment score; R, right; L, left; Trunk Sum score: sum of quantitative severity score in trunk.

^‡^,Spearman correlation.

^*^revealed P < 0.05, ^**^revealed P < 0.01.

A total of 7 patients with DM1 were analyzed using a Southern blotting method. There was no significant correlation between CTG repeat number and trunk sum score or CTG repeat number and total sum score.

## Discussion

In this study, muscle ultrasonography of LOPD demonstrated prominent involvement in the abdominal and a relative sparing in the distal extremities. The current result was consistent with the findings of muscle magnetic resonance imaging (MRI) or computed tomography (CT) in patients with LOPD at different stages of disease (23). In contrast, the abdominal muscle was less involved or sparing, and severe increases in muscle echogenicity of distal extremities in patients with DM1 was observed (24). There was a significant difference in echogenicity between patients with LOPD and patients with DM1 in abdominal muscles, biceps, and FDS/FDP. In addition, patients with late-onset Pompe revealed significantly lower thickness of lower abdominal muscles than that observed in patients with DM1. In the LOPD group, all abdominal muscle groups showed moderate to severe involvement, which correlated with clinical function. Muscle echogenicity and thickness could respond to a pathological change of muscle and functional impairment (23, 25).

Although qualitative (Heckmatt) ultrasound measures could classify muscle echogenicity, the image may be significantly influenced by the patient's age or body mass index (BMI) (21, 23, 26). Age, gender, and obesity status may influence muscle thickness and echogenicity (27–29). The Z score of quantitative ultrasound could be used to objectively investigate patient's muscle echogenicity using gray-scale analysis adjusted by healthy controls (7, 21, 28, 30, 31).

Trunk function has been found to contribute to maintaining good posture and functional movement (32). A previous study revealed increased fall risk in patients with more severe involvement of trunk muscles (13). Trunk muscle involvement has also been found to correlate with negative respiratory outcomes and balance function in LOPD and DM1 (10, 12). According to Vill et al., ultrasound could detect muscle abnormality in clinically affected muscles and demonstrated that clinical muscle strength was highly correlated with muscle abnormality in LOPD (6). Our study demonstrated that abdominal muscle strength and TIS score were correlated with abdominal muscle echogenicity. Total MRC sum score was not correlated with abdominal echogenicity but was significantly correlated with the total echo extremities sum score. In previous studies, the skeletal MRI study revealed prominent fatty infiltration of abdominal muscles in LOPD (5, 33). By contrast, prominent mild asymmetrical fatty infiltration was found in distal quadriceps, gastrocnemius, soleus, and tibialis anterior muscles in patients with DM1 (24). Overall, legs were more severely affected than thighs and pelvis as demonstrated in skeletal MRI (24). The current study revealed ultrasonography could also detect abdominal muscle abnormalities. This indicates that muscle ultrasonography is an inexpensive and simple tool to evaluate axial muscle impairment and clinical outcomes, particularly in patients with LOPD. Furthermore, the ultrasound echogenicity rather than thickness provides more significant differences between the two diseases and their correlation with muscle function.

Our study has several limitations, first, the late-onset Pompe group is small. However, in previous studies, the trunk muscles were all significantly involved and were highly correlated with the function scale. The previous studies also revealed that trunk muscle involvement was also very prominent in skeletal MRI (5, 33). Secondly, deeper muscles, such as iliopsoas and paraspinals, were not easily assessed by ultrasonography. However, further studies could resolve the problem by using a high-resolution ultrasound machine and apply it to other disease-specific myopathic patterns.

## Conclusion

Our study suggests that muscle ultrasound could be an efficient screening tool to assessing myopathic changes and disease-specific patterns that could help physicians with differential diagnosis of neuromuscular diseases. Trunk muscles could offer a preliminary opportunity for a differential diagnosis between LOPD and DM1. Muscle echography findings were also correlated with clinical and motor functions. TIS could also be used to investigate the trunk function of patients with myopathy.

## Data availability statement

The original contributions presented in the study are included in the article/Supplementary material, further inquiries can be directed to the corresponding author.

## Ethics statement

Ethical review and approval was not required for the study on human participants in accordance with the local legislation and institutional requirements. The patients/participants provided their written informed consent to participate in this study.

## Author contributions

P-CH and H-CK conceived, designed the study, and performed the experiments. P-CH performed all candidates' ultrasonography evaluations, analyzed the data, and then wrote the paper. J-EC performed the figures. All authors read and approved the final manuscript.

## Funding

This work was supported by grants CMRPG3J1751, CMRPG3K1871, and CMRPG3L1121 from Chang Gung Memorial Hospital, Taoyuan, Taiwan.

## Conflict of interest

The authors declare that the research was conducted in the absence of any commercial or financial relationships that could be construed as a potential conflict of interest.

## Publisher's note

All claims expressed in this article are solely those of the authors and do not necessarily represent those of their affiliated organizations, or those of the publisher, the editors and the reviewers. Any product that may be evaluated in this article, or claim that may be made by its manufacturer, is not guaranteed or endorsed by the publisher.

## Abbreviations

GAA: acid alpha-glucosidase
LOPD: late-onset Pompe disease
DM1: myotonic dystrophy type 1
BB: biceps brachii
FDS/FDP, flexor digitorium superficialis/flexor digitorium profundus, RF, rectus femoris, TA, tibialis anterior, RA, rectus abdominis, EO: external oblique
IO: internal oblique
TrA: transversus abdominis muscles
TIS: trunk impairment scale
TCT: trunk control test
MRC: Medical Research Council.
