# Supplementary material for: Ultrasonography of abdominal muscles: Differential diagnosis of late-onset Pompe disease and myotonic dystrophy type 1
Source: Front Neurol. 2022 Sep 6;13:944464. doi: 10.3389/fneur.2022.944464 (PMC9488967; doi:10.3389/fneur.2022.944464)
Supplement: Supplementary file 1 [file Data_Sheet_1.docx]

**Table S1 intra-rater and inter-rater correlation**

| The intra-rater and inter-rater reliability in quantitative muscle image | | |
| --- | --- | --- |
| Intraclass Correlation 1 | Low Bound | High Bound |
| 0.904 | 0.815 | 0.912 |
| Intraclass Correlation 2 |  |  |
| 0.892 | 0.720 | 0.961 |
| Interclass Correlation |  |  |
| 0.887 | 0.656 | 0.966 |
| The intra-rater and inter-rater reliability in muscle thickness | | |
| Intraclass Correlation 1 | Low Bound | High Bound |
| 0.987 | 0.961 | 0.995 |
| Intraclass Correlation 2 |  |  |
| 0.916 | 0.776 | 0.970 |
| Interclass Correlation |  |  |
| 0.975 | 0.948 | 0.988 |

**Table S2 Grading of muscle strength in abdominal for manual muscle test**

| Muscle test | Position | Movement | Resistance | Grades |
| --- | --- | --- | --- | --- |
| Trunk flexors | Supine  Leg flexion | Full trunk curl, then sit up  Full trunk curl, then sit up  Bilateral hand could touch the knee | Arms behind neck | 5 |
|  |  |  | Arms folded over chest | 4 |
|  |  |  | Arms along the body | 3 |
|  |  | Pelvic posterior tilt | Arms along the body | 2 |
|  |  |  | Only feel contraction in abdominal muscle | 1 |

| **Table S3 demographic and functional data in control group** | | | | | | |
| --- | --- | --- | --- | --- | --- | --- |
|  | Age | Muscle power of trunk (0-5) | MRC sum score (0- 120) | TCT (0-100) | TIS Total score (0-30) |  |
| Male |  |  |  |  |  |  |
| N=6 | 20-29 | 5 | 120 | 100 | 30 |  |
| N=6 | 30-39 | 5 | 120 | 100 | 30 |  |
| N=5 | 50-59 | 4.5 | 120 | 100 | 30 |  |
| Female |  |  |  |  |  |  |
| N=5 | 20-29 | 5 | 120 | 100 | 30 |  |
| N=7 | 30-39 | 4.7 | 120 | 100 | 30 |  |
| N=6 | 50-59 | 3 | 120 | 100 | 30 |  |

MRC, medical research council; TCT, trunk control test; TIS, trunk impairment score
